# Biochemical and Structural Studies of Conserved Maf Proteins Revealed Nucleotide Pyrophosphatases with a Preference for Modified Nucleotides

**DOI:** 10.1016/j.chembiol.2013.09.011

**Published:** 2013-11-21

**Authors:** Anatoli Tchigvintsev, Dmitri Tchigvintsev, Robert Flick, Ana Popovic, Aiping Dong, Xiaohui Xu, Greg Brown, Wenyun Lu, Hong Wu, Hong Cui, Ludmila Dombrowski, Jeong Chan Joo, Natalia Beloglazova, Jinrong Min, Alexei Savchenko, Amy A. Caudy, Joshua D. Rabinowitz, Alexey G. Murzin, Alexander F. Yakunin

**Affiliations:** 1 Department of Chemical Engineering and Applied Chemistry, University of Toronto, Toronto, ON M5S 3E5, Canada; 2 Structural Genomics Consortium, University of Toronto, Toronto, ON M5G 1L6, Canada; 3 Lewis-Sigler Institute for Integrative Genomics, Department of Chemistry, Princeton University, Princeton, NJ 08544, USA; 4 Donnelly Centre for Cellular and Biomolecular Research, University of Toronto, Toronto, ON M5S 3E1, Canada; 5 MRC Laboratory of Molecular Biology, Francis Crick Avenue, Cambridge CB2 0QH, UK

## Abstract

Maf (for multicopy associated filamentation) proteins represent a large family of conserved proteins implicated in cell division arrest but whose biochemical activity remains unknown. Here, we show that the prokaryotic and eukaryotic Maf proteins exhibit nucleotide pyrophosphatase activity against 5-methyl-UTP, pseudo-UTP, 5-methyl-CTP, and 7-methyl-GTP, which represent the most abundant modified bases in all organisms, as well as against canonical nucleotides dTTP, UTP, and CTP. Overexpression of the Maf protein YhdE in *E. coli* cells increased intracellular levels of dTMP and UMP, confirming that dTTP and UTP are the in vivo substrates of this protein. Crystal structures and site-directed mutagenesis of Maf proteins revealed the determinants of their activity and substrate specificity. Thus, pyrophosphatase activity of Maf proteins toward canonical and modified nucleotides might provide the molecular mechanism for a dual role of these proteins in cell division arrest and house cleaning.

## Highlights

• Maf proteins represent a family of nucleoside triphosphate pyrophosphatases
• Maf proteins hydrolyze the canonical nucleotides dTTP, UTP, and CTP
• Maf proteins are also active against m^5^UTP, m^5^CTP, pseudo-UTP, and m^7^GTP
• Maf structures reveal the molecular mechanisms of their substrate selectivity

## Graphical Abstract

**Figure:**
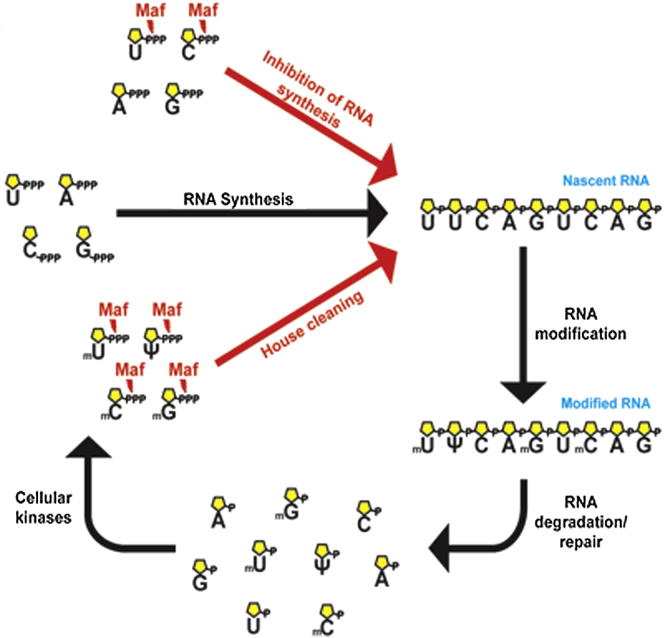

## Teaser

Tchigvintsev et al. show that Maf proteins are a family of nucleotide pyrophosphatases active against both canonical and modified nucleotides. This suggests that Mafs might have a dual role in cell division and in the prevention of the incorporation of modified nucleotides into cellular nucleic acids.

## Introduction

Global genome sequencing efforts have revealed millions of genes encoding unknown proteins for which there is no direct experimental proof of function (Galperin and Koonin, 2004; Hanson et al., 2010; Osterman and Overbeek, 2003; Roberts, 2004). “Unknown” and “hypothetical” proteins represent a large fraction (30% to 50%) of the genes in sequenced genomes and metagenomes, limiting our knowledge of these organisms and biology in general. Maf-like proteins constitute a large family of conserved unknown sequences found in bacteria, archaea, and eukaryotes (over 7,300 sequences in databases; Pfam PF02545 and IPR003697). The name Maf (for multicopy associated filamentation) was proposed in a previous genetic work, which demonstrated that the introduction of *maf* on a multicopy plasmid into *Bacillus subtilis* cells resulted in an inhibition of septation and extensive filamentation of cells (Butler et al., 1993). However, the inactivation of *maf* did not produce any apparent phenotype in *B. subtilis* cells, indicating that it is not required for cell division (Butler et al., 1993). The *B. subtilis* Maf protein is homologous to the *Escherichia coli* YhdE (previous name, OrfE; 46% sequence identity), and on the chromosome, both genes are associated with the shape-determining genes *mreBCD*. The morphogenetic complex MreBCD is responsible for the maintenance of rod cell morphology, and the *E. coli* MreB is also involved in chromosome segregation and nucleoid separation (Butler et al., 1993; Kruse et al., 2005; Wachi et al., 1991).

The crystal structure of the *B. subtilis* Maf (BSU28050) was published in 2000, and, to date, it represents the only experimental work with a purified Maf protein (Minasov et al., 2000). The structure revealed a structural fold similar to that found in ITPases and YjjX proteins, which are nucleotide pyrophosphatases hydrolyzing ITP, dITP, and XTP (Hwang et al., 1999; Savchenko et al., 2007; Zheng et al., 2005). Based on the subsequent analysis of BSU28050 structure, Maf proteins have been proposed to belong to a group of “house-cleaning” nucleotide hydrolyzing enzymes, which also include Nudix hydrolases, ITPases, dUTPases, and all-α nucleoside triphosphate pyrophosphatases (Bessman et al., 1996; Galperin et al., 2006; Moroz et al., 2005). The house-cleaning nucleotide pyrophosphatases hydrolyze various noncanonical nucleotides (dUTP, dITP, 8-oxo-dGTP, and 2-oxo-dATP), which can cause mispairing and mutation if incorporated into DNA. Thus, house-cleaning enzymes prevent the incorporation of noncanonical nucleotides into cellular DNA working in parallel with DNA repair proteins. The structural similarity of ITPases and *B. subtilis* Maf suggests that these proteins have a common evolutionary origin and that Maf proteins are likely to catalyze a similar chemical reaction; however, no nucleotide hydrolysis was demonstrated for BSU28050 (Minasov et al., 2000). The structure of BSU28050 in complex with dUTP (Protein Data Bank [PDB] code 1EXC) also revealed that the dUTP base is located on the protein surface and makes limited contacts with the protein (Minasov et al., 2000). However, a subsequent analysis of this structure pointed to a large pocket equivalent to the base recognition site of ITPases, suggesting that Maf proteins can bind and hydrolyze the nucleotide substrates like ITPases (Galperin et al., 2006).

Interest in Maf proteins was revived by a recent genetic work on the *B. subtilis* BSU28050, which indicated that it is involved in the cell division arrest associated with DNA transformation and repair (Briley et al., 2011). Following DNA damage or DNA transformation, cells need to inhibit cell division to allow time for DNA repair or integration of transforming DNA. In competent *B. subtilis* cells, cell division is blocked because the traffic ATPase ComGA inhibits the polymerization of the tubulin-like protein FtsZ (Haijema et al., 2001). In *B. subtilis*, *maf* is a competence-induced gene whose product (1) is localized near the cell poles; (2) interacts with ComGA, DivIVA, and FtsW; and (3) blocks septation during the escape from competence (when the transformed cells resume growth) (Briley et al., 2011). The traffic ATPase ComGA is essential for the binding of transforming DNA to the competent cell surface and its uptake inside the cell (Chung and Dubnau, 1998), whereas DivIVA is a scaffold protein that helps to localize other proteins (e.g., the division inhibitor MinC/MinD) to cell division sites or polar regions (Lenarcic et al., 2009). This work demonstrated that Maf is responsible for cell division inhibition in the absence of ComGA, suggesting that Maf functions as an additional (backup) system in the delay of cell division (Briley et al., 2011). What is intriguing about Maf is that this biochemically uncharacterized protein is conserved in all kingdoms of life, suggesting that it represents a general molecular mechanism of the inhibition of cell division (Hamoen, 2011).

Here, we present the results of the biochemical, structural, and mutational studies of six Maf proteins from prokaryotic and eukaryotic organisms, including *E. coli*, *Salmonella typhimurium*, *B. subtilis*, *Saccharomyces cerevisiae*, and humans. We have demonstrated that the two subfamilies of Maf proteins (YhdE and YceF) have nucleoside triphosphate pyrophosphatase activity against both canonical (dTTP, UTP, and CTP) and modified (5-methyl-UTP, pseudo-UTP, 5-methyl-CTP, and 7-methyl-GTP) nucleotides. Overexpression of the *E. coli* Maf protein YhdE increased the intracellular concentration of dTMP and UMP. Crystal structures of the human ASMTL-Maf domain, BSU28050, and the *E. coli* YceF revealed their active site, which was further characterized by site-directed mutagenesis and substrate docking. We propose that nucleoside triphosphate pyrophosphatase activity of Maf proteins against canonical and noncanonical nucleotides might represent a molecular mechanism for a dual role of these proteins in cell division arrest and in preventing the incorporation of modified nucleotides into cellular nucleic acids (house cleaning).

## Results

### Two Subfamilies of Maf Proteins: YhdE and YceF

Analysis of available sequenced genomes indicates that *maf* genes are present in most organisms, including bacteria, archaea, and eukaryotes. Most proteobacteria, *Planctomycetes*, and many eukaryotes (plants, protozoa) contain two genes encoding Maf proteins (e.g., YhdE and YceF in *E. coli*), whereas one *maf* gene is present in other bacteria, archaea, and humans. The *E. coli* Maf proteins YhdE and YceF share low sequence similarity to each other (35% sequence identity), and they are more similar (44% to 70% sequence identity) to the orthologous sequences in other proteobacteria. Sequence analysis of two groups of Maf proteins revealed the conservation of the six charged or polar residues in all members, which therefore represent the signature Maf motif (S-R-E-K-D-K): Ser10, Arg13, Glu33, Lys52 (in YhdE and YceF), Asp70 (Asp69 in YceF), and Lys82 (Lys81 in YceF) (Figure S1 available online). The main differences between the YhdE and YceF subfamilies include the YhdE Arg12 (replaced by an aromatic residue in YceF proteins), Thr71 (replaced by Gln70 in YceF), and Gln153 (replaced by Glu154 in YceF) (Figure S1). Thus, these residues represent the subfamily-specific motifs for the two main groups of Maf proteins: YhdE (R-T-Q) and YceF (W-Q-E). Most Maf proteins can be assigned to either the YhdE subfamily or the YceF subfamily on the basis of the presence of these sequence motifs, e.g., the *Trypanosoma brucei* TB927.1.3280 (YhdE-like) and Tb11.01.5890 (YceF-like). In many eukaryotic Maf proteins, including the human ASMTL, the N-terminal YhdE-like Maf domain is covalently fused to another domain encoding a predicted methyltransferase protein, whereas in some archaeal genomes (e.g., *Staphylothermus marinus*), the Maf gene is associated with a methyltransferase gene, suggesting a functional relationship between these proteins. Thus, sequence analysis of Maf proteins from sequenced genomes suggests that there are two main subfamilies of these proteins, YhdE and YceF, which might have different substrate preferences.

### Nucleotide Pyrophosphatase Activity of Maf Proteins

**Figure 1:**
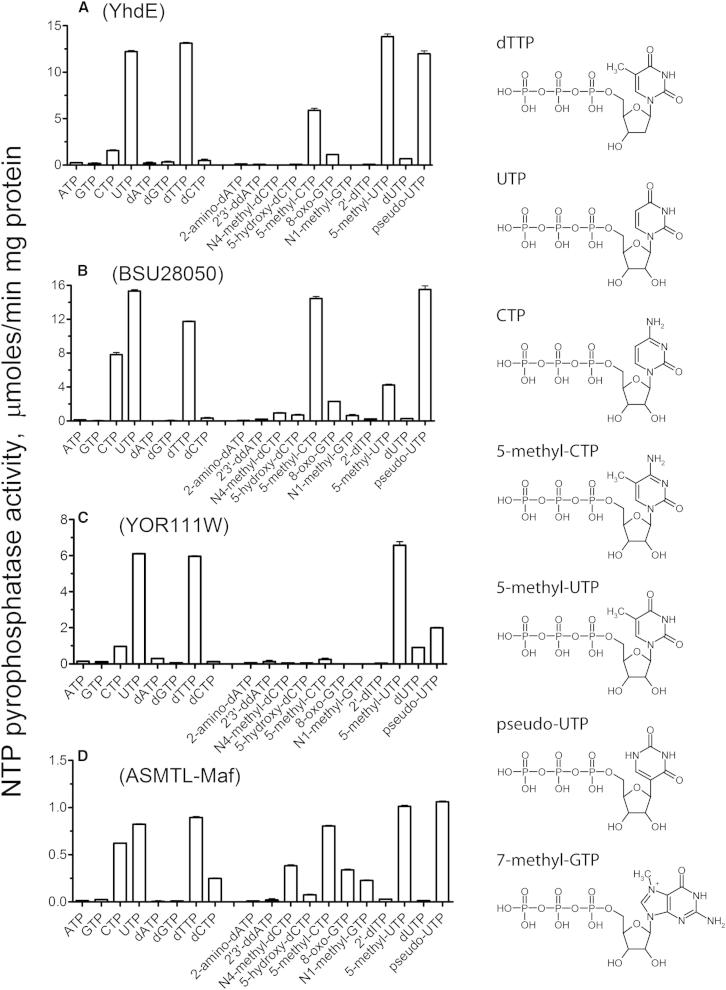
Nucleoside Triphosphate Pyrophosphatase Activity of Purified Maf Proteins: Hydrolysis of Canonical and Modified Nucleotides The graphs show specific activities of (A) YhdE, (B) BSU28050, (C) YOR111W, and (D) ASMTL-Maf against canonical and modified nucleotides (the left and right parts of the graph, respectively). The reaction mixtures contained 0.2 mM substrate, 2 mM Co^2+^, and 0.1–0.5 μg of enzyme. Results are means ± SD from at least two independent determinations. The molecular structures of positive Maf substrates are shown on the right side of the graph. See also Figure S2 and Table S1.

The genes encoding four Maf proteins from the YhdE subfamily (the *E. coli* YhdE, *B. subtilis* BSU28050, *S. cerevisiae* YOR111W, and human ASMTL Maf domain, 1–239 amino acids [aa]) and two Maf proteins from the YceF subfamily (the *E. coli* YceF and *S. typhimurium* STM1189) were overexpressed in *E. coli*, and the recombinant proteins were purified to at least 95% homogeneity. General enzymatic assays (Kuznetsova et al., 2005) revealed no presence of phosphatase, phosphodiesterase, esterase, protease, dehydrogenase, oxidase, or nuclease activities in these proteins (data not shown). Since the previous bioinformatic analysis of Maf proteins suggested that they might have nucleoside triphosphate pyrophosphatase activity (Galperin et al., 2006), purified Maf proteins were tested for this activity using the canonical ribo- and deoxyribonucleoside triphosphates as substrates (Figure 1). Whereas both YceF-like proteins were inactive against these substrates (30–90 nmol/min per milligram of protein), the four YhdE-like Maf proteins (*E. coli* YhdE, BSU28050, YOR111W, and ASMTL-Maf) showed significant metal-dependent pyrophosphatase activity against pyrimidine nucleoside triphosphates dTTP, UTP, and CTP (Figure 1). These proteins showed no activity without the addition of a divalent cation, whereas Co^2+^ and Mg^2+^ (0.5–10 mM) supported substrate hydrolysis in both the pyrophosphatase-coupled and high-performance liquid chromatography (HPLC)-based assays (Figure S2).

Screening of Maf proteins for pyrophosphatase activity against a set of 57 noncanonical or modified nucleoside triphosphates (Table S1) revealed that the YhdE-like Maf proteins (*E. coli* YhdE, BSU28050, YOR111W, and ASMTL-Maf) also exhibited high pyrophosphatase activity against 5-methyl-UTP (m^5^UTP or riboTTP), pseudouridine triphosphate (pseudo-UTP or Ψ), and 5-methyl-CTP (m^5^CTP), whereas the two YceF proteins (from *E. coli* and *S. typhimurium*) were active toward 7-methyl-GTP (m^7^GTP) (Figure 1; Table 1). These nucleotides represent the most abundant naturally occurring modifications found in all RNA species (tRNA, rRNA, mRNA, and small nuclear and nucleolar RNAs) in all kingdoms of life, which are formed by posttranscriptional modification of specific canonical bases in RNA by specialized enzymes (methylases and pseudouridine synthases) (Cantara et al., 2011). Thus, the two different subfamilies of Maf proteins have retained nucleotide pyrophosphatase activity but have evolved different substrate selectivities toward canonical or modified nucleotides.

Purified Maf proteins exhibited high affinity to their substrates in vitro. The Michaelis-Menten constant, K_M_, of the YhdE-like proteins toward canonical pyrimidine nucleotides varied in a micromolar range: 32.7–53.0 μM for dTTP; 29.7–72.4 μM for UTP; and it was lower (to a lesser extent for ASMTL-Maf) with modified nucleotides (4.7–47.2 μM) (Table 1). Both YceF proteins also showed high affinity to m^7^GTP (32.8 μM for YceF and 30.9 μM for STM1189) (Table 1). Hydrolysis of m^7^GTP by the *E. coli* YceF was inhibited in a dose-dependent manner by unmodified nucleotides, with 50% reduction at 80–100 μM (GTP, ATP, UTP), which is higher than its K_M_ to m^7^GTP (32.8 μM). The range of K_M_ of Maf proteins with canonical pyrimidine nucleoside triphosphates is within the range of their known intracellular concentrations in prokaryotic and eukaryotic cells (from 1.5 μM to 8 mM) (Bennett et al., 2009; Ditzelmüller et al., 1983). Thus, the enzymatic activity of Maf proteins can potentially contribute to transformations of intracellular nucleotides in vivo.

### Effect of Maf Overexpression on the *E. coli* Nucleotide Pools

**Figure 2:**
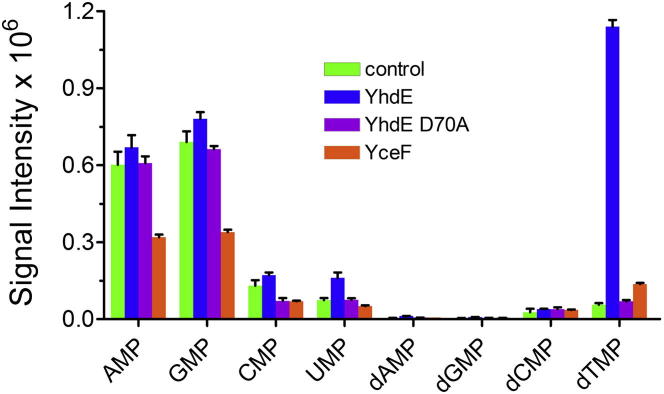
Effect of the Maf Overexpression on the *E. coli* Nucleoside Monophosphate Metabolome LC-MS analysis of the intracellular nucleoside monophosphates in the *E. coli* strains containing the empty expression plasmid or overexpressing the wild-type or inactive Maf proteins. Results are means ± SD from at least two independent determinations. See also Figure S3.

To determine if the overexpression of Maf proteins in *E. coli* cells affects their nucleotide pools, we performed a liquid chromatography-mass spectrometry (LC-MS) analysis of nucleotides extracted from the cells overexpressing the wild-type YhdE or YceF proteins. The overexpression of the wild-type YhdE in *E. coli* cells increased the dTMP level more than 10 times and the UMP pool two times compared to the control strain containing an empty plasmid, whereas the levels of other nucleotides (including nucleoside triphosphates) showed no significant changes (Figure 2; Figure S3). It is known that the intracellular nucleoside triphosphate pools are sensitive to preparative steps making it difficult to capture true changes in pool sizes (Buckstein et al., 2008; Kimball and Rabinowitz, 2006). The *E. coli* cells overexpressing the inactive YhdE D70A mutant protein (discussed later) or wild-type YceF showed the same nucleotide levels as in the control strain (Figure 2). The levels of modified nucleotides (m^5^UTP, m^5^UMP, pseudoUTP, pseudoUMP, m^5^CTP, m^5^CMP, m^7^GTP, and m^7^GMP) in the *E. coli* samples were below the detection limit of the LC-MS protocol used. Thus, the results of LC-MS analysis of *E. coli* metabolome suggest that dTTP and UTP are the in vivo substrates of YhdE, supporting our in vitro activity results.

### Crystal Structure of Maf Proteins: ASMTL-Maf, BSU28050, and YceF

**Figure 3:**
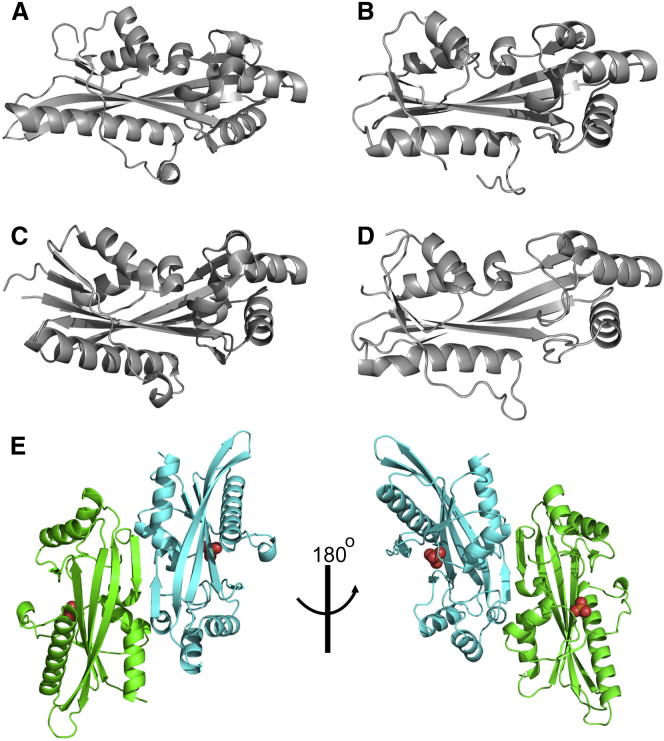
Crystal Structures of Maf Proteins (A–D) Overall structures of the protomers are: (A) ASMTL-Maf (PDB code 2P5X), (B) BSU28050 (PDB code 4HEB), (C) Tb-Maf1 (PDB code 2AMH), and (D) YceF (PDB code 4JHC). (E) The ASMTL-Maf dimers (two orientations related by 180° rotation). The ribbon diagrams of two monomers are in green and cyan, and the bound phosphate molecule is shown as balls with oxygen in red and phosphorus in orange. See also Table S2.

ASMTL-Maf, BSU28050, and YceF were crystallized, and their structures were solved at 2.0 Å (ASMTL-Maf), 2.26 Å (BSU28050), or 1.85 Å (YceF) resolution by molecular replacement using the structure of BSU28050 (PDB code 1EX2) as a model (Table S2). The unpublished structure of the *T. brucei* Maf protein Tb11.01.5890 (Tb-Maf1) was determined by the Structural Genomics of Pathogenic Protozoa Consortium and submitted to the PDB in 2005 (PDB code 2AMH). The protomer structures of the four Maf proteins revealed a large β sheet containing seven β strands (β1 to β7) with two α-helical subdomains on one side and one long α helix on another side (Figure 3; Figure S1). The four Maf proteins have highly similar structures, with the differences occurring mainly at the insertion/deletion sites in their sequence alignment (Figure S1). The position of the active site in Maf proteins is indicated by the phosphate or sulfate molecules, which are bound in the large cavity between the two helical subdomains in the structures of ASMTL-Maf, Tb-Maf1, and BSU28050 (Figure 3).

Analysis of the crystal contacts using the quaternary prediction server PISA suggested that the Maf proteins form dimers through multiple interactions between residues located on their C-terminal regions (155–183 in BSU28050, 181–202 in ASMTL-Maf, and 155–193 in YceF). In the ASMTL-Maf structure, two protomers form a dimer via the formation of a common large β sheet through the interaction between their β6 strands (Figure 3B; Figure S1). This oligomeric state is consistent with the results of our gel-filtration analysis, which showed that ASMTL-Maf exists as a dimer in solution (observed molecular mass, 44.5 kDa; predicted monomer molecular mass, 28.3 kDa), whereas YhdE exists as a mixture of dimers and tetramers (observed molecular mass, 47.2 and 101 kDa, respectively; predicted monomer molecular mass, 24 kDa) and YceF as a mixture of monomers and dimers (observed molecular mass, 24.8 and 50 kDa, respectively; predicted molecular mass, 24.5 kDa). Previously, BSU28050 has also been reported to exist as a dimer in solution (Minasov et al., 2000).

A Dali search (Holm and Rosenström, 2010) for the ASMTL-Maf structural homologs identified the Maf proteins from *B. subtilis* (PDB code 1EX2; Z score, 24.8–25.9; root-mean-square deviation [rmsd], 1.7–1.9 Å) and *T. brucei* (PDB code 2AMH; Z score, 23.8; rmsd, 1.8 Å), as well as several ITPases: TM0159 from *Thermotoga maritima* (PDB code 1VP2; Z score, 11.8; rmsd, 2.8 Å), MJ0226 from *Methanococcus jannaschii* (PDB code 1B78; Z score, 10.4; rmsd, 2.9 Å), and RdgB from *E. coli* (PDB code 2Q16; Z score, 10.3; rmsd, 2.7 Å) (Hwang et al., 1999; Savchenko et al., 2007). Similar results were obtained for Tb-Maf1 and YceF. High structural similarity between the Maf proteins and ITPases suggests a close evolutionary relationship of these protein families.

### Site-Directed Mutagenesis and Catalytic Mechanism of Maf Proteins

**Figure 4:**
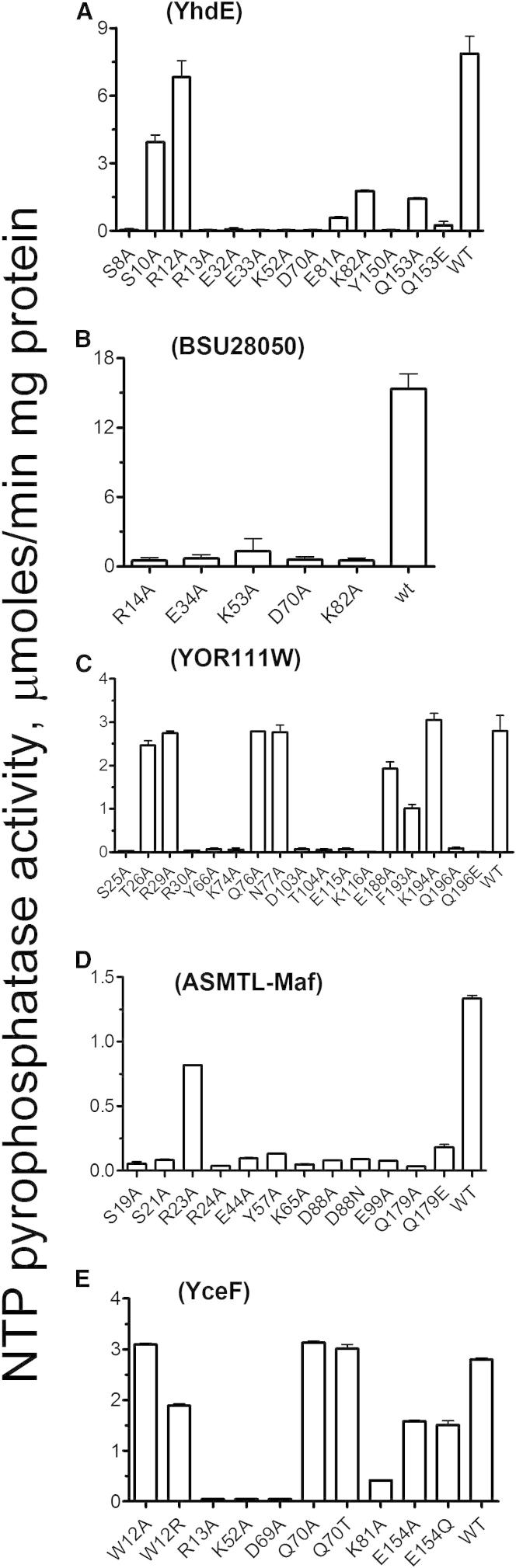
Site-Directed Mutagenesis of Maf Proteins: Nucleotide Pyrophosphatase Activity of Purified Mutant Proteins (A–E) Mutant proteins shown are (A) YhdE, (B) BSU28050, (C) YOR111W, (D) ASMTL-Maf, and (E) YceF. The assays contained 0.3 mM UTP in (A) through (D) or 0.2 mM m^7^GTP (E), 2 mM Co^2+^ in (A) and (C), 5 mM Co^2+^ in (B) and (D), or 0.2 mM Co^2+^ (E) and 0.03–0.5 μg of protein. Results are means ± SD from at least two independent determinations. See also Figures S1 and S5.

The crystal structures of four Maf proteins (BSU28050, YceF, *T. brucei* Maf1, and ASMTL-Maf) provide insights into the molecular mechanisms of activity and substrate selectivity of these enzymes. A structure-based sequence alignment of these proteins identified 14 conserved charged, polar, and aromatic residues, most of which are located in the large cavity formed by the main beta sheet and alpha helices accommodating the bound phosphate molecules (Figure S1). Most of these residues were mutated to Ala in YhdE, BSU28050, YOR111W, ASMTL-Maf, and YceF, and the mutant proteins were overexpressed in *E. coli* and purified. Enzymatic assays with purified proteins revealed that most of them showed greatly reduced activity, indicating that many active-site residues are important for activity (Figure 4). In contrast, mutagenesis of the nonconserved residues of YOR111W (Thr26, Gln76, Asn77, Glu188, Phe193, and Lys194) produced mutant proteins with activities and catalytic efficiencies similar to that of the wild-type protein (Figure 4; Table 1).

**Figure 5:**
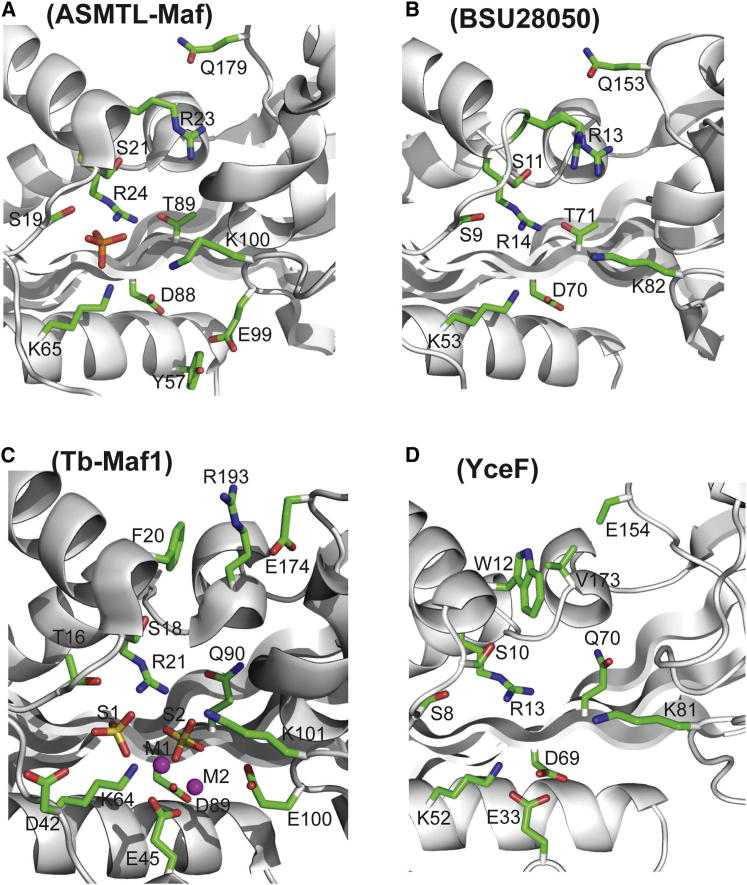
Active Site of Maf Proteins Close-up view of the active site of (A) ASMTL-Maf with bound phosphate, (B) BSU28050, (C) Tb-Maf1 with bound sulfates and metal ions, and (D) YceF. The protein side chains are shown as green sticks along a protein ribbon (gray). In (A), the bound phosphate is shown as orange sticks with red indicating oxygen. In (C), the bound sulfates (S1 and S2) are shown as yellow sticks with red indicating oxygen, and the bound metal ions (M1 and M2) are shown as the magenta balls. In the YceF active site in (D), the side chain of Glu154 is incompletely modeled because it was disordered. See also Figures S1 and S4–S6.

In Maf proteins and structurally similar ITP pyrophosphatases, the side chain of the predicted catalytic Asp (ASMTL-Maf Asp88; YOR111W Asp103) interacts with the conserved Lys (ASMTL-Maf Lys65, 2.9 Å; YOR111W Lys74), possibly stabilizing the unprotonated state of Asp (Gutteridge and Thornton, 2005) (Figure 5). A pH-rate profile analysis of YOR111W produced a parabolic curve with two logarithmic acid dissociation constants (pK_a_) values, 6.8 and 8.2, suggesting that at least two enzyme residues are involved in catalysis (Figure S4). Since most active-site carboxylates have pK_a_ values >5.5 (Forsyth et al., 2002), the YOR111W pK_a_ of 6.8 might be associated with the deprotonation of the catalytic Asp103, whereas the pK_a_ of 8.2 might be associated with the protonation of Lys74. Therefore, we propose that the unprotonated Asp88 of ASMTL-Maf (Asp70 in YhdE, Asp103 in YOR111W, Asp70 in BSU28050, and Asp69 in YceF) functions as a general base coordinating a water molecule to produce a nucleophilic hydroxide ion, which attacks the alpha-phosphate of the substrate (Figure 5). This is supported by the complete loss of activity in the ASMTL-Maf D88A (and D88N), YhdE D70A, YOR111W D103A, BSU28050 D70A, and YceF D69A mutant proteins (Figure 4). The structure of the inactive YceF D69A protein showed no changes in the position of the side chains of other residues in the active sites, suggesting that the loss of activity in this protein is caused by the replacement of the catalytic Asp69 by Ala (Figure S5). Near the catalytic Asp residue, there are three conserved positive residues (Arg14, Lys53, and Lys82 in BSU28050 and Arg24, Lys65, and Lys100 in ASMTL-Maf) (Figure 5) whose side chains have a trigonal orientation in the active site and are arranged similarly to the triphosphate binding site of the *E. coli* dITPase RdgB (Lys13, Lys53, and Arg87) (Savchenko et al., 2007). The structures of ASMTL-Maf and Tb-Maf1 also showed the presence of additional electron densities located in the large cavity, which were modeled as a phosphate or two sulfate molecules (Figure 5). In ASMTL-Maf, the phosphate molecule is located close to the side chains of the conserved Ser19 (2.4 Å), Arg24 (2.6 Å), Lys65 (2.6 Å), and Lys100 (4.0 Å), and it probably mimics the position of the substrate γ-phosphate (Figure 5). In Tb-Maf1, sulfate molecule-1 is close to the side chains of Thr16 (3.4 Å) and Arg21 (3.8 Å), whereas the sulfate molecule-2 is bound by the side chains of Arg21 (2.8 Å) and Lys64 (3.1 Å), possibly representing the positions of the substrate γ- and α-phosphates, respectively. In BSU28050, the pyrophosphate-like molecule is coordinated by the side chains of conserved Ser9 (2.7 Å), Arg14 (2.8 Å and 2.9 Å), and Lys53 (3.0 Å), and its position is similar to that expected for the β-γ-pyrophosphate part of a nucleoside triphosphate substrate (Figure S6). Alanine replacement mutagenesis of the predicted triphosphate binding residues in ASMTL-Maf (Arg24, Lys65, Lys100), YOR111W (Arg30, Lys74, Lys116), YhdE (Arg13, Lys52, Lys82), BSU28050 (Arg14, Lys53, Lys82), and YceF (Arg13, Lys52, Lys81) produced inactive proteins (Figure 4), confirming that these residues play an important role in activity of Maf proteins and suggesting that they are likely to be involved in the coordination of the triphosphate part of the substrate. Of note, a recent work with *B. subtilis* has demonstrated that the K53A mutant Maf protein (BSU28050) is deficient in complementation of a *maf* deletion (Briley et al., 2011). Thus, our results imply that the nucleotide pyrophosphatase activity of Maf proteins appears to be important for their function in vivo.

The structure of Tb-Maf1 (PDB code 2AMH) also revealed the presence of two metal ions (Mn^2+^) bound in the active site. One metal ion is coordinated by the oxygen atoms of two sulfate molecules and Glu45 side chain (2.4 Å), whereas the second Mn^2+^ is coordinated by two oxygens of one sulfate molecule (2.3 Å and 2.6 Å) and by the side chain oxygens of the conserved Glu45 (2.2 Å) and catalytic Asp89 (2.2 Å) (Figure 5C). The Tb-Maf1 Glu45 is conserved in all Maf proteins (Figure S1), and alanine replacement mutagenesis of homologous residues in YhdE (E33A), BSU28050 (E34A), and ASMTL-Maf (E44A) produced proteins with low activity (Figure 4). Therefore, we propose that, in the active site of Maf proteins, the catalytic metal ion is bound to the substrate phosphate oxygens and to the side chains of the conserved N-terminal Glu and catalytic Asp (Glu44 and Asp88 in ASMTL-Maf), whereas the second metal ion is bound to the substrate triphosphate part contributing to its charge neutralization.

### Structural Basis of the Substrate Selectivity of Maf Proteins

The structures of Maf proteins suggest that their nucleoside binding pocket is located next to the triphosphate binding site between the two helical lobes (Figures 3 and 5). Since the purified Maf proteins failed to cocrystallize with substrates, we performed a knowledge-based substrate docking of different nucleoside triphosphate molecules using the triphosphate model from the two structures of the ITPase-ITP complexes (PDB codes 2Q16 and 2J4E) as the starting point. The α- and γ-phosphates of ITP superposed well with the positions of bound phosphate or sulfate molecules in the Maf structures (BSU28050, ASMTL-Maf, and Tb-Maf1), whereas the nucleoside part of substrates was docked into the nucleoside-binding pocket using the rotations around its covalent bonds.

**Figure 6:**
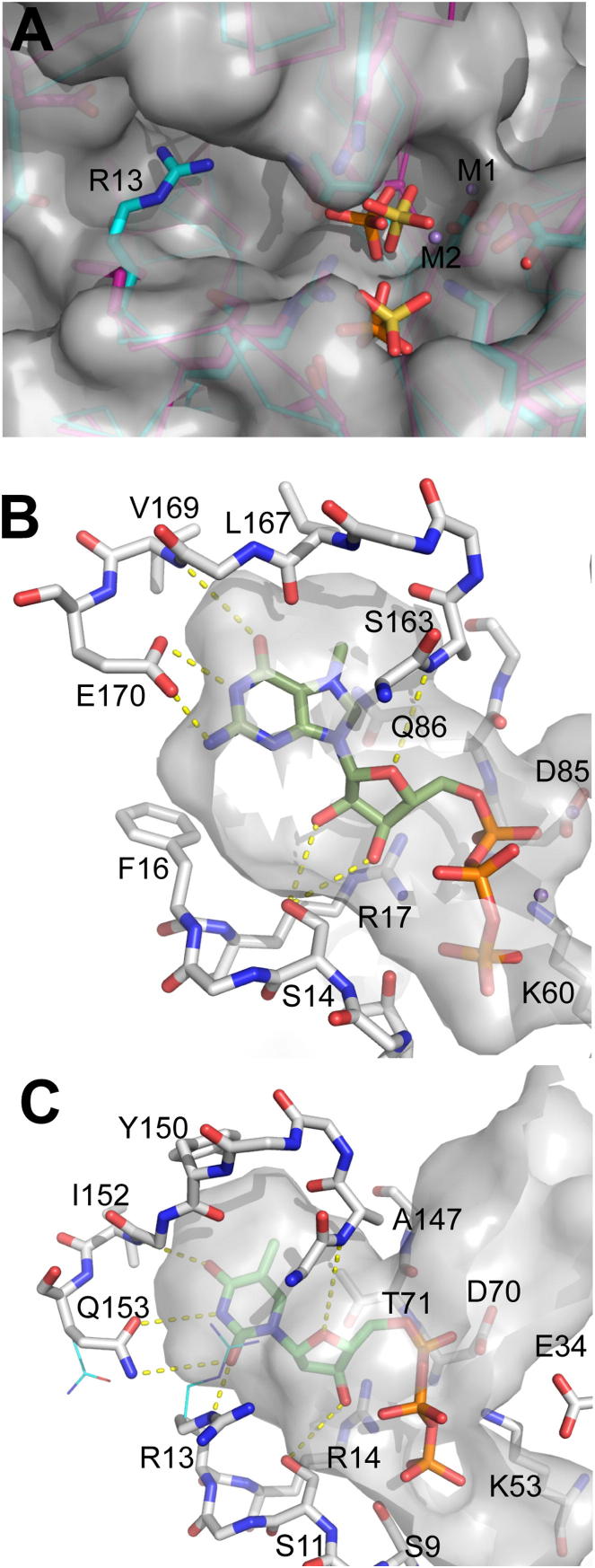
Structural Basis of Different Substrate Specificity of Two Maf Subfamilies (A) Structural superposition of the substrate binding sites of BSU28050 (cyan; YhdE subfamily) and Tb-Maf1 (magenta; YceF subfamily). Side chains of conserved residues, as well as phosphate (orange) and sulfate (yellow) ions bound to BSU28050 and Tb-Maf1, respectively, are shown as sticks. Also shown are the bound Mn^2+^ ions (M1 and M2) and the surface of the Tb-Maf1. In BSU28050, the side chain of the conserved Arg13 is located in the base-binding pocket, but it can be flipped away without altering the main chain conformation. (B) The base-binding pocket of the Tb-Maf1 (YceF subfamily) with manually docked m^7^GTP (dark green carbons). The predicted substrate specificity determinants are the Val173 main chain amide, Gln90, and Glu174 (labeled and also shown with potential H-bonds). (C) The base-binding pocket of the BSU28050 (YhdE subfamily) with manually docked dTTP (light green carbons). The predicted substrate specificity determinants are Arg13, Thr71, and Gln153 (labeled and also shown with potential H-bonds). The side chain conformations of Arg13 and Gln153 have been changed to different rotamers compared to the original structure shown in (A). See also Figure S7.

The two structures of YceF-like Maf proteins (Tb-Maf1 and YceF) appear to display an open conformation of the active site, which can accommodate a nucleoside group of the substrate without any conformational change in the binding pocket. In contrast, in the structures of the YhdE-like Maf proteins BSU28050 and ASMTL-Maf, the nucleoside-binding pocket is partially occluded by the guanidine group of an Arg residue (Arg13 in BSU28050, Arg12 in YhdE, Arg29 in YOR111W, and Arg23 in ASMTL-Maf) (Figure 6A). As shown in Table 1, both substrate affinities and activities of the YhdE R12A, YOR111W R29A, and ASMTL-Maf R23A mutant proteins against dTTP were more or less close to that of the wild-type proteins. The new structure of BSU28050 (PDB code 4HEB) shows that the side chain of the homologous Arg13 can adopt two alternative conformations with its guanidine group positioned in or out of the pocket. The Tb-Maf1 contains a phenylalanine residue at this site (Phe16) whose side chain points away from the pocket, whereas the Glu170 side chain is oriented toward the pocket and can participate in base recognition. This Glu residue is conserved in YceF (Glu154) and other YceF-like Maf proteins, making it the signature residue-3 of the YceF subfamily motif (Figure S1B). In the YceF structure, the Glu154 side chain is partly disordered, suggesting local structural flexibility (Figure 5D). It should be noted that the YceF E154A mutant protein retained the wild-type level of substrate affinity and catalytic activity (Table 1). The nucleoside binding pocket of Tb-Maf1 can readily accommodate a purine nucleoside, with the Glu170 side chain and the backbone NH-group of Val169 providing a specific recognition of guanine base through the formation of three H-bonds and enough space to accommodate the N7 methyl group of m^7^G (Figure 6B). The Tb-Maf1 Gln86 (Gln70 in YceF) resides at the bottom of the pocket, providing the side chain-main chain H-bonds to the C end of the α6 helix and thus determining the size of the guanine recognition site (Figure S1B). This is in line with a greatly reduced substrate affinity of the YceF Q70A mutant protein (Table 1). Since the Gln86 side chain is at the H-bond distance from the N7 group, it may also be involved in the discrimination against the canonical nucleotide GTP by promoting an alternative, nonproductive binding mode for unmodified guanine nucleotide and preventing its hydrolysis (Figure 6B).

The YhdE-like Maf proteins contain a conserved glutamine residue (Gln153 in BSU28050) at the position equivalent to that of the Tb-Maf1 Glu170, which represents the signature residue-3 of the YhdE subfamily motif and an apparent structural determinant for substrate selectivity of this subfamily (Figure S1A). However, in the structures of BSU28050 and ASMTL-Maf, the side chains of Gln153 and Gln179, respectively, are oriented slightly away from the substrate-binding pocket; therefore, they cannot contribute to base recognition in this conformation (Figure 6C). After a minor repositioning of the Arg13 side chain out of base pocket and the Gln153 side chain toward it, the binding pocket of BSU28050 can readily accommodate pyrimidine nucleoside triphosphates in a productive mode (Figure 6C). The proposed mode of thymine base recognition by the YhdE-like Maf proteins is analogous to the predicted binding of m^7^GTP by YceF-like proteins (Figures 6B and 6C). The side chain amide group of Gln153 and the main chain NH group of Ile152 can form three H-bonds to the O2, N3, and O4 groups of thymine, whereas the repositioned guanidine group of Arg13 can make an additional H-bond to the thymine O2 (Figure 6C). In the BSU28050 nucleoside binding pocket, the C5 methyl group of thymine occupies a position, which is structurally equivalent to the position of the N7 methyl group of m^7^G in the YceF pocket (Figures 6B and 6C). The uracil and pseudouracil bases will make essentially the same H-bonds as thymine, with the remaining small cavity (at the position of the C5 methyl group) occupied by a conserved water molecule forming an additional H-bond to the pseudouracil N1 group (Figure S7A). The m^5^C base is isosteric to thymine and is predicted to fit into the pocket in the same way (Figure S7B). In contrast, the predicted position of the unmodified cytosine base is notably different as it can form different H-bond interactions in the absence of the C5 methyl group. This results in the N4 group of cytosine slipping into the vacant m^5^C site (Figures 1 and S7C). This would explain the reduced activity against CTP compared to m^5^CTP for the YhdE-like Maf proteins (except for YOR111W, which exhibits low activity against both modified and unmodified cytosine triphosphates). Finally, both YhdE- and YceF-like Maf proteins use a similar mode for binding the ribose or deoxyribose groups of substrate with a conserved Ser residue (Ser10 in YhdE, Ser11 in BSU28050, and Ser14 in Tb-Maf1) serving as the main sugar recognition determinant, which forms H-bonds to both 2′-OH and 3′-OH groups (Figure 6). The YhdE S10A mutant protein exhibited both lower substrate affinity and catalytic activity compared to the wild-type protein (Table 1). In addition, the models explain a slight preference of Maf proteins toward ribonucleotides by the formation of additional H-bond(s) to the 2′-OH group. This additional interaction may compensate for a loose fit in the base-recognition pocket. Indeed, the YhdE-like Maf proteins exhibit comparable activities against both dTTP (which has a better fit but fewer H-bonds) and UTP (which has a worse fit but more H-bonds) (Figure 1). In contrast, the preferred substrate m^5^UTP (which has a better fit and more H-bonds) consistently shows the lowest K_M_ values and the highest catalytic rate constant (k_cat_)/K_M_ values (Table 1).

The proposed models of recognition of the modified nucleotide bases by Maf proteins present a mechanism of discrimination between the methylated (substrate) and nonmethylated (nonsubstrate) molecules. It is well known that the presence of a methyl group in a molecule can introduce steric hindrance in the enzyme active site and prevent productive binding of methylated molecules (Helt et al., 2008; Jen-Jacobson et al., 1996). In contrast, the presence of a methyl-group size cavity in the active site is unlikely to abolish the binding of an unmethylated molecule (a nonsubstrate). We propose that, due to a loose fit, a nonsubstrate (unmethylated) molecule slips into an alternative, unproductive binding mode. Such a slippage can be promoted by the formation of new specific interactions (e.g., H-bonds) between the enzyme and nonsubstrate. The “slippage” mechanism implements the negative principle of protein design, according to which the main role of methyl group in the substrate molecule is not the stabilization of the formation of a productive enzyme-substrate complex but the prevention of the nonproductive binding of substrate in the active site.

## Discussion

Thus, the biochemical and structural studies of six Maf proteins from different organisms have revealed two subfamilies of new enzymes with the metal-dependent nucleoside triphosphate pyrophosphatase activity against both canonical and noncanonical pyrimidine nucleotides (YhdE-like proteins) or m^7^GTP (YceF-like proteins) (Table 1). Crystal structures revealed the active sites of Maf proteins and molecular mechanisms of preference for canonical (dTTP and UTP) and modified nucleotides (pseudo-UTP, m^5^CTP, m^5^UTP, and m^7^GTP), as well as a mechanism of enzyme preference for methylated bases. The preference of Maf proteins for modified ribonucleotides in vitro suggests that, in vivo, these enzymes are likely to monitor the ribonucleotide pool and prevent unspecific incorporation of modified bases into cellular RNAs. In some organisms, Maf proteins can function in parallel with the UTPase subfamily of Nudix hydrolases, which have been shown to be active against 5-methyl-UTP (riboTTP) and UTP in vitro (Xu et al., 2003).

**Figure 7:**
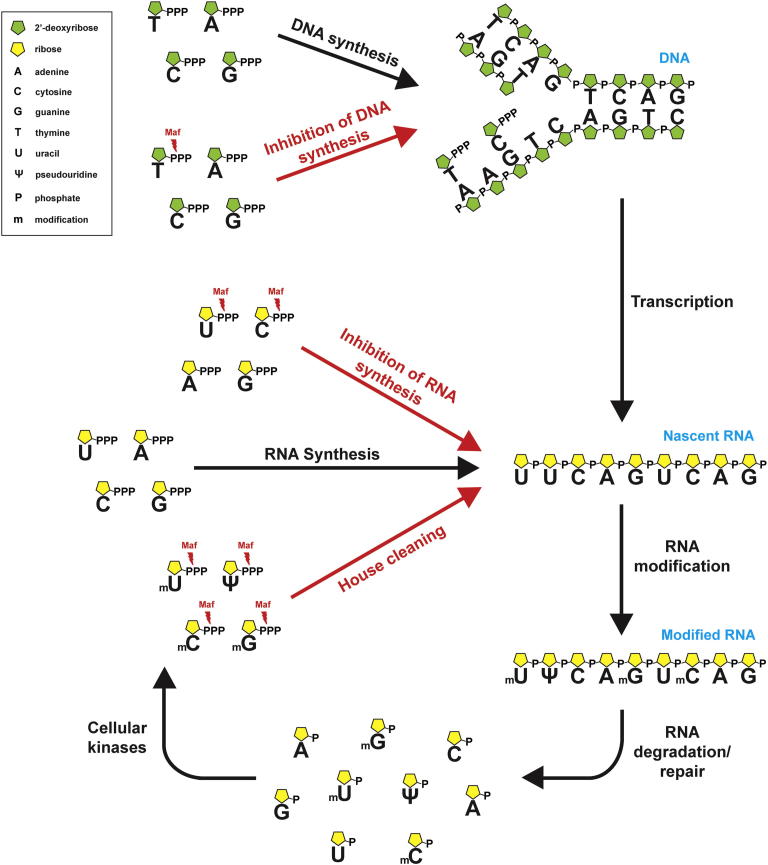
Proposed Role of Maf Proteins in House Cleaning and Cell Division Arrest Top: cell division arrest. Nucleoside triphosphate pyrophosphatase activity of Maf proteins from the YhdE subfamily reduces the intracellular pool of dTTP, resulting in inhibition of DNA synthesis/replication and delaying cell division. Bottom: house cleaning. In the cells, the modified RNA bases are produced by specific modifying enzymes, and they enter the cellular nucleotide pool as nucleoside monophosphates during RNA degradation or repair. The modified nucleosides are then phosphorylated by various nucleotide kinases to the respective triphosphates (Ψ, m^5^CTP, m^5^UTP, and m^7^GTP), whose incorporation into RNA is prevented by Maf proteins.

Currently, over 110 RNA or DNA base modifications are known, which are introduced by specific modifying enzymes and are important for gene expression, translation, DNA repair, stress response, and host-pathogen interactions (Cantara et al., 2011). The pseudouridine (Ψ), 5-methyluridine (m^5^U), 5-methylcytidine (m^5^C), and 7-methylguanosine (m^7^G) are the most abundant modified nucleosides found in all organisms, with Ψ being the most abundant modified nucleoside in both prokaryotic and eukaryotic RNAs. They are synthesized at the RNA level by different modifying enzymes including pseudouridine synthases and methylases, which catalyze the site-specific isomerisation or methylation of RNA bases (Gustafsson et al., 1996; Koonin, 1996) (Figure 7). It is expected that degradation and repair of cellular nucleic acids containing modified bases will produce the respective nucleoside monophosphates (m^5^CMP, m^5^dCMP, m^5^UMP, and m^7^GMP), which can be converted into triphosphates and incorporated into newly synthesized nucleic acids by RNA polymerases (Bessman et al., 1958; Goldberg and Rabinowitz, 1963; Kahan and Hurwitz, 1962) (Figure 7). Thus, hydrolysis of the modified nucleoside triphosphates by Maf proteins in vivo might reduce their incorporation into cellular nucleic acids and diminish their potential mutagenic effects.

With canonical nucleotides as substrates, the YhdE-like Maf proteins showed high affinity to dTTP, UTP, and CTP with a micromolar K_M_ (25.0 to 105.9 μM), which is within the range of known intracellular concentrations of these nucleotides in prokaryotic and eukaryotic cells (1.5 μM to 8 mM) (Bennett et al., 2009; Ditzelmüller et al., 1983). Thus, in vivo, Maf proteins can potentially slow down the synthesis of both DNA and RNA. Our metabolome analysis confirmed that dTTP and UTP are the in vivo substrates for the *E. coli* YhdE and, potentially, for other YhdE-like proteins. We postulate that the pyrophosphatase activity of Maf proteins against the canonical pyrimidine nucleotides (Figure 1) might represent one of the molecular mechanisms of the inhibition of cell division by Maf proteins. This is consistent with the results of recent work on *B. subtilis*, which revealed that the BSU28050 K53A mutant protein is deficient in the complementation of a *maf* deletion, as observed by the lack of restoration of the *comGA::Tn917* filamentous phenotype (Briley et al., 2011). Our in vitro assays with the purified mutant proteins BSU28050 (K53A), YhdE (K52A), YceF (K52A), YOR111W (K74A), and ASMTL-Maf (K65A) demonstrated complete, or almost complete, inactivation of nucleotide pyrophosphatase activity in all proteins (Figure 4), supporting our hypothesis that this activity plays an important role in the in vivo function of Maf proteins.

Thus, the nucleoside triphosphate pyrophosphatase activity of Maf proteins against canonical and noncanonical nucleotides might represent a molecular mechanism for a dual role of Maf proteins in the inhibition of cell division and in preventing the incorporation of modified nucleotides into cellular nucleic acids. Considering that Maf proteins from different organisms have similar structural and biochemical properties, including substrate profiles, their enzymatic activity might represent a general mechanism contributing to cell division arrest and house cleaning in all kingdoms of life.

## Significance

**Maf proteins represent a large family of conserved unknown sequences found in bacteria, archaea, and eukaryotes. Twenty years ago, it was shown that they appear to function as a negative regulator of cell division, but the biochemical activity and molecular mechanism of their function remained unknown. In 2006, a bioinformatic work proposed that Maf proteins might also contribute to “house cleaning” by preventing the incorporation of modified, mutagenic nucleotides into newly synthesized nucleic acids. Using a combination of enzymology, metabolomics, site-directed mutagenesis, protein crystallography, and structural analysis, we have demonstrated that Maf proteins represent a family of nucleoside triphosphate pyrophosphatases active against both canonical (dTTP, UTP, and CTP) and modified (pseudo-UTP, 5-methyl-UTP, 5-methyl-CTP, 7-methyl-GTP) nucleotides. These modified nucleotides represent the most abundant naturally occurring modifications present in all RNA species in all organisms. Using liquid chromatography-mass spectrometry, we confirmed that dTTP and UTP are the in vivo substrates for the *E. coli* Maf protein YhdE. Crystal structures and site-directed mutagenesis of Maf proteins revealed the determinants of their substrate specificity and proposed a mechanism of enzyme preference for methylated substrates. Nucleotide pyrophosphatase activity of Maf proteins toward canonical and modified nucleotides might represent a molecular mechanism for the dual role of these proteins in the inhibition of cell division and in the prevention of the incorporation of modified nucleotides into cellular nucleic acids.**

## Experimental Procedures

### Protein Expression, Purification, and Mutagenesis

The genes encoding Maf proteins from *E. coli* (YhdE, P25536; YceF, P0A729), *S. typhimurium* (YceF, P58627), *B. subtilis* (BSU28050, Q02169), *S. cerevisiae* (YOR111W, Q99210), and human (ASMTL Maf domain, 1–239 aa; O95671) were cloned, purified, and mutated as described elsewhere (Kuznetsova et al., 2005).

### Enzymatic Assays

Purified Maf proteins were screened for the presence of several general enzymatic activities (phosphatase, phosphodiesterase, esterase, protease, and dehydrogenase) or nuclease activity against short, single-stranded DNA and RNA using the protocols described elsewhere (Beloglazova et al., 2008; Kuznetsova et al., 2005). Pyrophosphatase activity against the commercially available canonical and modified nucleoside triphosphates was determined by measuring the P_i_ release in the presence of inorganic pyrophosphatase (from baker’s yeast) and 2 mM Co^2+^ at 30°C (BSU28050 and YOR111W) or 37°C (YhdE, YceF, STM1189, and ASMTL-Maf) as described elsewhere (Tchigvintsev et al., 2011). The dependence of the Maf proteins’ activity on divalent metal cations was determined with 0.2 mM UTP as substrate in the presence of saturating concentrations of cations (5 mM Mg^2+^, 1 mM Mn^2+^, 0.5 mM Zn^2+^, or 2 mM Co^2+^), using the pyrophosphatase-based assay or HPLC with a Varian Pursuit C18 column (Varian ProStar HPLC system) (Tchigvintsev et al., 2011).

### Metabolome Analysis

*E. coli* cells (BW25113) containing the pCA24N protein expression vector with no insert or expressing the wild-type or mutant YhdE or YceF were grown overnight in Luria broth (LB) medium (37°C). On the next day, 200 μl of overnight culture was inoculated into 20 ml of fresh LB medium for each data point and grown at 37°C to an optical density 600 (OD_600_) of 0.4. Induction of protein expression was conducted at 37°C for 30 min by the addition of 1 mM IPTG. At an OD_600_ of 0.5, the cultures were rapidly filtered on to 25 mm (0.4 μm) nylon membranes using a 10-place vacuum manifold (Amersham) and the filter-bound cells were quenched by submersion in 1 ml of −20°C extraction solvent (40:40:20 acetonitrile/methanol/water containing 0.1 M formic acid). The samples for LC-MS analysis were prepared as described elsewhere (Lu et al., 2010; Rabinowitz and Kimball, 2007) and analyzed using an Exactive orbitrap mass spectrometer equipped with an electrospray ionization source and a Hypersil Gold C18 column (50 mm × 2.1 mm, 1.9 μm particle size; Thermo Scientific).

### Protein Crystallization

Purified human ASMTL-Maf protein (5.7 mg/ml) was crystallized using the sitting drop vapor diffusion method at 20°C by mixing 1 μl of protein solution with 1 μl of reservoir solution containing 15% polyethylene glycol (PEG) 3350, 0.1 M succinic acid (pH 7.0). Purified BSU28050 (10 mg/ml) was crystallized using the hanging drop vapor diffusion method at 20°C by mixing 1 μl of the protein solution with 1 μl of the reservoir solution containing 2 M sodium formate, 0.1 M Bis-Tris propane, pH 7.5. Crystals of the *E. coli* YceF (16 mg/ml) were grown at 22°C using the sitting drop vapor diffusion method. Prior to set-up, the purified YceF was pretreated with papain (1/10, v/v) (Dong et al., 2007), and the crystals were grown in the presence of 0.2 M sodium dihydrogen phosphate and 20% PEG 3350 (wild-type YceF) or 0.1 MES (pH 6.0) and 20% PEG 10000 (YceF D69A).

### Data Collection, Structure Determination, and Refinement

X-ray diffraction data for the human ASMTL-Maf, BSU28050, and YceF crystals were collected at 100K using the Rigaku FRE High Brilliance X-Ray Generator with R-AXIS IV detector. Data were processed using the HKL-2000 suite (Otwinowski and Minor 1997). The structures of human ASMTL-Maf, BSU28050, and YceF were solved by molecular replacement using MOLREP (Vagin and Teplyakov 1997). The crystal structure of BSU28050 (PDB code 1EX2) was used as the search model. ARP/wARP (Perrakis et al., 1999) was used for automatic model building. REFMAC (Murshudov et al., 1997) was used for structure refinement. The graphics program COOT (Emsley and Cowtan, 2004) was used for model building and visualization. Crystal diffraction data and refinement statistics for three protein structures are presented in Table S2.

## Figures and Tables

**Table 1:** Kinetic Parameters of the Wild-Type and Mutant Maf Proteins

Protein	Variable substrate	K_M_ (μM)	k_cat_ (s^−1^)	k_cat_/K_M_ (s^−1^M^−1^)
YhdE				
WT	dTTP	53.0 ± 2.0	15.4 ± 0.3	2.9 × 10^5^
WT	UTP	69.1 ± 3.1	15.2 ± 0.5	2.2 × 10^5^
WT	CTP	105.9 ± 10.8	4.9 ± 0.2	4.6 × 10^4^
WT	m^5^UTP	32.0 ± 2.2	18.2 ± 0.7	5.7 × 10^5^
WT	m^5^CTP	44.8 ± 2.2	9.2 ± 0.2	2.1 × 10^5^
WT	pseudoUTP	47.2 ± 1.5	10.5 ± 0.2	2.2 × 10^5^
S10A	dTTP	78.9 ± 3.5	4.5 ± 0.1	5.7 × 10^4^
R12A	dTTP	40.9 ± 2.0	5.5 ± 0.1	1.3 × 10^5^
K82A	dTTP	36.1 ± 2.0	0.8 ± 0.1	2.2 × 10^4^
BSU28050				
WT	dTTP	50.6 ± 2.1	11.6 ± 0.2	2.3 × 10^5^
WT	UTP	72.4 ± 5.2	4.9 ± 0.2	6.8 × 10^4^
WT	CTP	25.0 ± 0.8	7.1 ± 0.1	2.8 × 10^5^
WT	m^5^UTP	4.7 ± 0.2	2.4 ± 0.1	5.1 × 10^5^
WT	m^5^CTP	7.8 ± 0.2	8.5 ± 0.1	1.1 × 10^6^
WT	pseudoUTP	5.7 ± 0.3	7.2 ± 0.2	1.3 × 10^6^
YOR111W				
WT	dTTP	47.4 ± 2.3	5.7 ± 0.1	1.2 × 10^5^
WT	UTP	29.7 ± 1.4	2.6 ± 0.1	8.8 × 10^4^
WT	CTP	72.0 ± 2.6	1.2 ± 0.02	1.7 × 10^4^
WT	m^5^UTP	13.0 ± 0.3	2.2 ± 0.1	1.7 × 10^5^
WT	pseudoUTP	20 ± 0.4	5.3 ± 0.1	2.7 × 10^5^
T26A	dTTP	72.5 ± 5.2	4.1 ± 0.1	5.7 × 10^4^
R29A	dTTP	39.4 ± 0.8	6.7 ± 0.1	1.7 × 10^5^
Q76A	dTTP	60.7 ± 4.7	3.7 ± 0.1	6.1 × 10^4^
N77A	dTTP	56.9 ± 3.7	3.1 ± 0.1	5.4 × 10^4^
E188A	dTTP	42.9 ± 2.1	1.4 ± 0.1	3.3 × 10^4^
F193A	dTTP	62.3 ± 2.0	3.4 ± 0.1	5.5 × 10^4^
K194A	dTTP	52.1 ± 3.5	4.1 ± 0.1	7.9 × 10^4^
ASMTL-Maf				
WT	dTTP	32.7 ± 3.4	0.7 ± 0.1	2.1 × 10^4^
WT	UTP	41.2 ± 9.3	0.7 ± 0.1	1.7 × 10^4^
WT	CTP	17.4 ± 0.8	0.8 ± 0.02	4.6 × 10^4^
WT	dCTP	22.8 ± 1.6	0.3 ± 0.01	1.3 × 10^4^
WT	m^5^UTP	16.1 ± 1.5	1.5 ± 0.1	9.3 × 10^4^
WT	m^5^CTP	39.4 ± 6.5	3.7 ± 0.3	9.4 × 10^4^
WT	pseudoUTP	18.7 ± 3.8	2.5 ± 0.2	1.3 × 10^5^
WT	8-oxo-GTP	10.7 ± 0.6	0.2 ± 0.01	1.9 × 10^4^
WT	N4-m-dCTP	10.3 ± 0.7	0.3 ± 0.01	2.9 × 10^4^
R23A	dTTP	27.7 ± 1.0	1.6 ± 0.1	5.8 × 10^4^
YceF				
WT	m^7^GTP	32.8 ± 3.1	1.2 ± 0.1	3.7 × 10^4^
W12A	m^7^GTP	217.2 ± 42.3	6.4 ± 1.1	2.9 × 10^4^
Q70A	m^7^GTP	173.3 ± 11.5	5.0 ± 0.3	2.9 × 10^4^
E154A	m^7^GTP	79.0 ± 17.4	2.1 ± 0.4	2.7 × 10^4^
STM1189				
WT	m^7^GTP	30.9 ± 1.2	1.5 ± 0.1	4.9 × 10^4^

The assays were performed in the presence of saturating concentrations of Co^2+^ (2 mM) at 30°C (for BSU28050 and YOR111W) or 37°C (for other proteins). WT, wild type. See also Figure S4.

## Accession Numbers

The atomic coordinates have been deposited at the PDB, with accession codes 2P5X (ASMTL-Maf), 4HEB (BSU28050), 4JHC (wild-type YceF), and 4LU1 (the YceF D69A mutant).
