# Supplementary material for: Biochemical and Structural Studies of Conserved Maf Proteins Revealed Nucleotide Pyrophosphatases with a Preference for Modified Nucleotides
Source: Chem Biol. 2013 Nov 21;20(11):1386–98. doi: 10.1016/j.chembiol.2013.09.011 (PMC3899018; doi:10.1016/j.chembiol.2013.09.011)

Chemistry & Biology, Volume *20*

Supplemental Information

Biochemical and Structural Studies of Conserved Maf Proteins Revealed Nucleotide Pyrophosphatases with a Preference for Modified Nucleotides

Anatoli Tchigvintsev, Dmitri Tchigvintsev, Robert Flick, Ana Popovic, Aiping Dong, Xiaohui Xu, Greg Brown, Wenyun Lu, Hong Wu, Hong Cui, Ludmila Dombrowski, Jeong Chan Joo, Natalia Beloglazova, Jinrong Min, Alexei Savchenko, Amy A. Caudy, Joshua D. Rabinowitz, Alexey G. Murzin, and Alexander F. Yakunin

Table S1, related to Figure 1. List of modified nucleoside triphosphates used for screening of purified Maf proteins.

1. 2',3'-ddATP	30. 5-propynyl-2'-deoxycytidine-5'-triphosphate
2. 2'-deoxyuridine-5'-triphosphate	31. 6-azauridine-5'-triphosphate
3. 2-amino-2'-dATP	32. 6-chloropurine-2'-deoxyriboside-5'-triphosphate
4. 2-amino-6-chloropurineriboside-5'-tri- phosphate	33. 6-chloropurineriboside-5'-triphosphate
5. 2-amino-6-Cl-purine-2'-deoxyriboside-triphosphate	34. 6-mercaptopurine-riboside-5'-triphosphate
6. 2-amino-ATP	35. 6-methylthio-ITP
7. 2-aminopurine-2'-deoxyriboside-triphosphate	36. 7-deaza-2'-deoxyguanosine-5'-triphosphate
8. 2'-dITP	37. 7-methyl-GTP
9. 2'-dUTP	38. 8-[(4-amino)butyl]-amino-ATP
10. 2'-O-methyl-GTP	39. 8-[(6-amino)hexyl]-amino-GTP
11. 3'-O-methyl-ATP	40. 8-bromo-GTP
12. 3'-O-methyl-GTP	41. 8-iodo-GTP
13. 5-aminoallyl-2'-deoxycytidine-5'-triphosphate	42. 8-oxo-2'-deoxyadenosine-5'-triphosphate
14. 5-aminoallylcytidine-5'-triphosphate	43. 8-oxo-2'-deoxyguanosine-5'-triphosphate
15. 5-bromo-2'-deoxycytidine-5'-triphosphate	44. 8-oxo-GTP
16. 5-bromo-CTP	45. 8-oxo-GTP
17. 5-bromocytidine-5'-triphosphate	46. gamma-aminophenyl-ATP
18. 5-bromo-UTP	47. N1-methyl-GTP
19. 5-fluoro-UTP	48. N2-methyl-2'-deoxyguanosine-5'-triphosphate
20. 5-hydroxymethyl dCTP	49. N4-methyl-2'-dCTP
21. 5-iodo-2'-deoxyuridine-5'-triphosphate	50. N6-(4-amino)butyl-ATP
22. 5-iodo-CTP	51. N6-methyl-2'-deoxyadenosine-5'-triphosphate
23. 5-iodo-dUTP	52. N6-methyladenosine-5'-triphosphate
24. 5-iodouridine-5'-triphosphate	53. N6-methyl-ATP
25. 5-methyl-2'-deoxycytidine-5'-triphosphate	54. O6-methyl-2'-deoxyguanosine-5'-triphosphate
26. 5-methyl-CTP	55. O6-methyl-GTP
27. 5-methyl-dCTP	56. pseudouridine-5'-triphosphate
28. 5-methyl-UTP (aka riboTTP)	57. xanthosine 5'-triphosphate
29. 5-OH-2'-dCTP	

Table S2, related to Figure 3. Data collection and refinement statistics for the crystal structures of ASMTL-Maf, BSU28050, and YceF.

	ASMTL-Maf	BSU28050	YceF wt	YceF D69A
**PDB Code**	2P5X	4HEB	4JHC	4LU1
**Data collection**				
Space group	$P2_12_12$	$P2_12_12_1$	$P2_12_12_1$	$P2_12_12_1$
Cell dimensions				
a, *b*, *c* (Å)	84.8, 117.0, 51.5	61.5, 86.0, 95.0	50.4,52.9,128.9	50.7,52.8,129.3
α, β, γ (°)	90, 90, 90	90, 90, 90	90, 90, 90	90, 90, 90
Resolution (Å)[a]	50.00-2.00 (2.07-2.00)	50.00-2.26 (2.30-2.26)	50.00-1.85 (1.88-1.85)	50.00-1.92 (1.95-1.92)
R_{merge}	10.2 (92.6)	10.7 (88.4)	4.3(58.6)	6.6(64.4)
$I/\sigma I$	14.4 (2.0)	19.7 (1.9)	37.4(2.0)	30.4(1.8)
Completeness (%)	99.7 (99.3)	99.9 (99.7)	99.9(99.7)	99.3(95.1)
Redundancy	6.9 (6.6)	7.3 (7.3)	7.6(5.7)	
**Refinement**				
Resolution (Å)	25.00 – 2.00	25.7-2.26	31.8-1.85	33.4-1.92
Number of reflections	35334	24200	29318	26175
R_{work}/ R_{free}	20.1/25.3	21.5/25.0	19.3/24.7	19.9/24.9
Number of atoms				
Protein	3190	2773	2907	2837
Phosphate	10			
Water	333	89	191	139
B-factors (Å^2)				
Protein	22.5	51.0	31.1	34.9
Phosphate	54.1			
Water	37.8	46.7	35.3	39.2
R.m.s. deviations				
Bond lengths (Å)	0.02	0.01	0.013	0.008
Bond angles (º)	1.4	1.1	1.1	1.272
**Ramachandran plot**				
Favored (%)	97.0	98.1	98.0	97.3
Additionally allowed (%)	3.0	1.9	2.0	2.7
Disallowed (%)	0.0	0.0	0.0	0.0

[a] Values in parentheses are for the highest-resolution shell.

Figure S1, related to Figures 4, and 5 . Structure-based sequence alignment of two sub-families of Maf proteins. (**A**), YhdE sub-family. (**B**), YceF sub-family. The secondary structure elements are shown above and below the alignment. Residues conserved in all aligned proteins are boxed with the Maf motif residues highlighted in orange, whereas the signature residues for the YhdE and YceF sub-families are highlighted in green and yellow, respectively. The residues mutated in this work are marked with black triangles and numbered. The proteins compared are: (A), *E. coli* YhdE (P25536), *B. subtilis* BSU28050 (Q02169), *Pyrococcus furiosus* PF0216 (Q8U476), *Methanosarcina barkeri* Mbar_A1652, *S. cerevisiae* YOR111W (Q99210), human ASMTL-Maf (O95671); (B), *E. coli* YceF (P0A729), *Salmonella typhimurium* YceF (STM1189, P58627), *Dictyostelium dsicoideum* (Q54TC5), *Pseudomonas aeruginosa* PA2972 (Q9HZN2), and *T. brucei* Tb11.01.5890 (Q382A9).

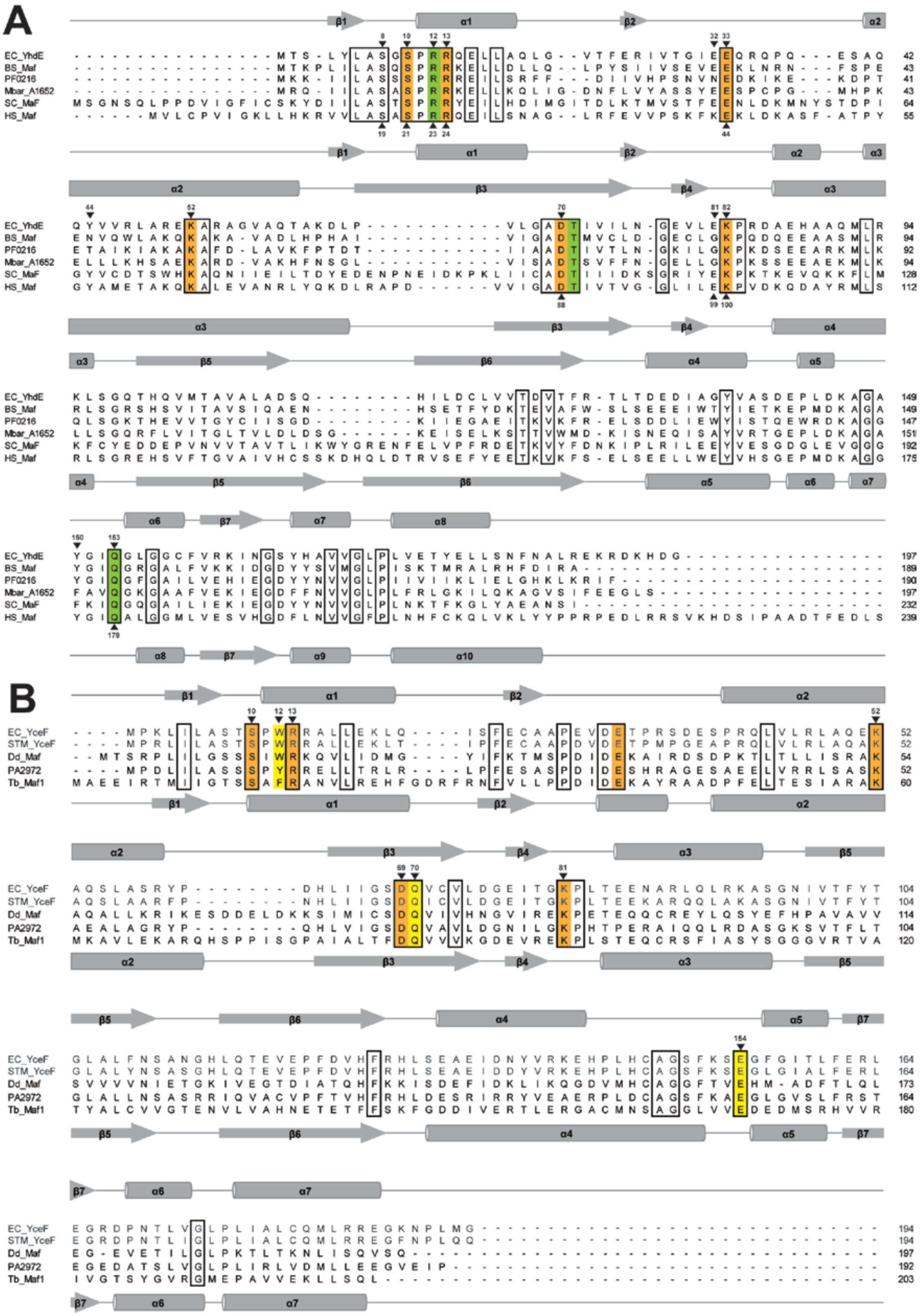
A
EC_YhdE
BS_Maf
PF0216
Mbar_A1652
SC_MaF
HS_Maf
B
EC_YceF
STM_YceF
Dd_Maf
PA2972
Tb_Maf1

Figure S2, related to Figure 1. Effect of divalent metal ions on pyrophosphatase activity of purified Maf proteins. Metal ion profiles of purified Maf proteins were analyzed using two different assays: a pyrophosphatase-coupled method (**A, B, C**) and an HPLC-based method (**D, E, F**). (**A, B, C**), Hydrolysis of UTP (0.2 mM) by YhdE (A, 0.5 μg), YOR111W (B; 0.1 μg), or ASMTL-Maf (C; 0.5 μg) was analyzed in the presence of saturating concentrations of Mg^{2+} (5 mM), Mn^{2+} (1 mM), Zn^{2+} (0.5 mM), or Co^{2+} (2 mM). (**D, E, F**), Hydrolysis of UTP (10 mM) by YhdE (D, 10 μg), YOR111W (E, 10 μg), or ASMTL-Maf (F, 10 μg) in the presence of different metal ions (as in A, B, C). The HPLC assay data are presented as a relative activity related to the activity of enzyme in the presence of Co^{2+}. Results are means ± S.D. from at least two independent determinations.

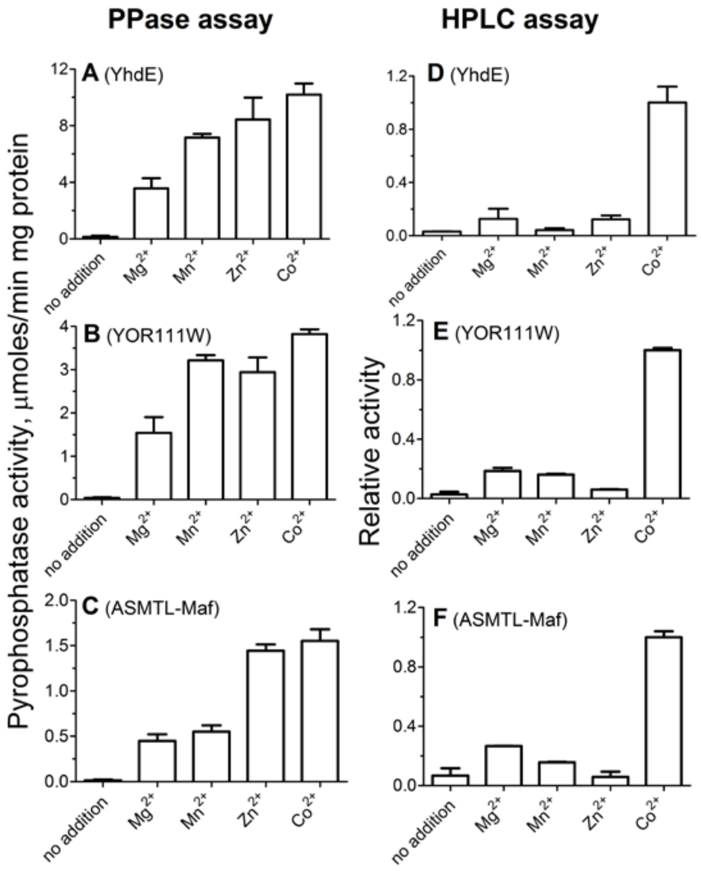

Figure S3, related to Figure 2. LC-MS analysis of ATP, GTP, and CTP in the *E. coli* cells over-expressing the wild type YhdE and YceF, as well as the inactive YhdE D70A mutant proteins. The *E. coli* BW25113 strain containing an empty protein expression plasmid pCA24N was used as a control. Results are means ± S.D. from at least two independent determinations.

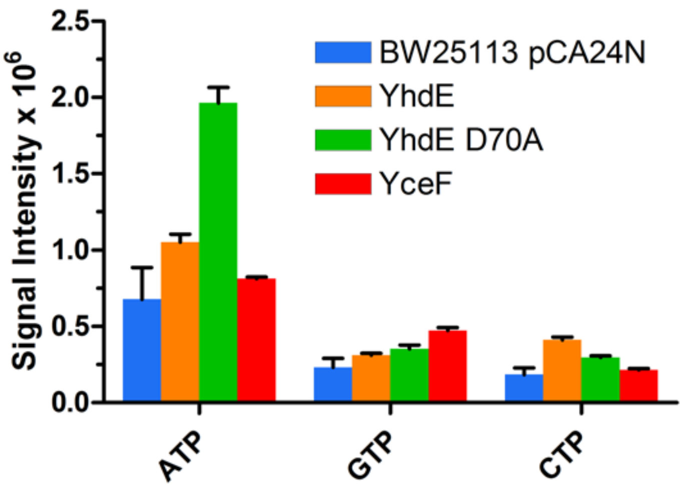

Figure S4, related to Figure 5 and Table 1. A pH-rate profile analysis of YOR111W: hydrolysis of dTTP. The reaction V_{max} was determined at the indicated pH values, and the curve was fitted as described in Segel I.H. Enzyme Kinetics (Wiley Classic Library edition 1993, pp. 884-926) using the following equation $V_{max}(pH) = (V_{max})^{max} / (1 + [H^+]/K_{a1} + K_{a2}/[H^+])$. $(V_{max})^{max}$ is the pH-independent value of V_{max}; H is hydrogen ion concentration; K_{a1} and K_{a2} are acid dissociation constants corresponding to pK values on the acid and basic side of the curve, respectively. The following buffers were used: MES (pH 5.5 – 6.5), HEPES (pH 7.0 – 8.0), and TAPS (pH 8.5 – 9.0). Results are means ± S.D. from at least two independent determinations.

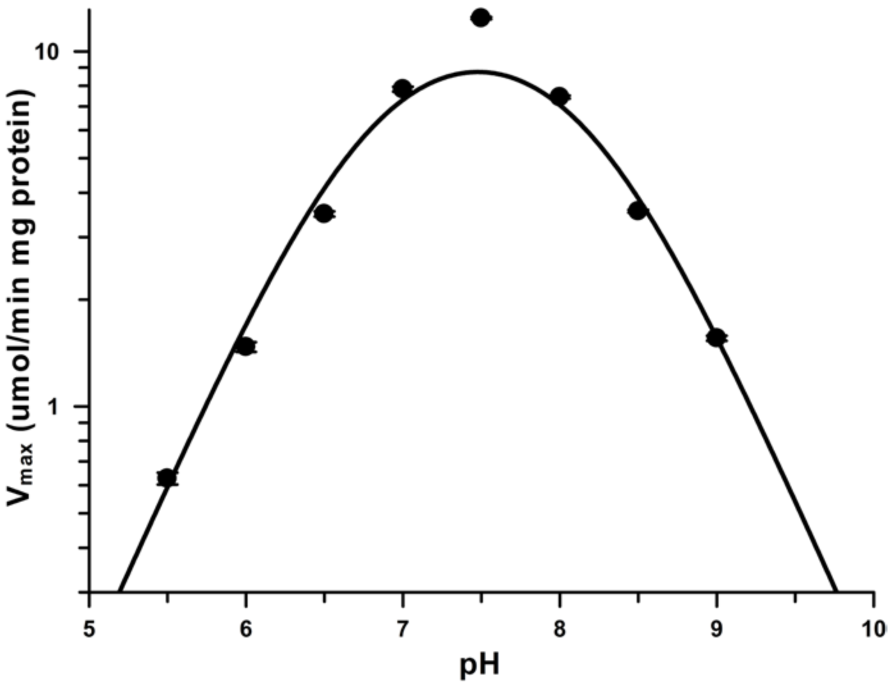

Figure S5, related to Figures 4 and 5. Structural superimposition of the wild type and D69A mutant YceF proteins. The protein ribbons and side chains are colored in different colours: yellow (wild type, PDB code 4JHC) and orange (D69A, PDB code 4LU1).

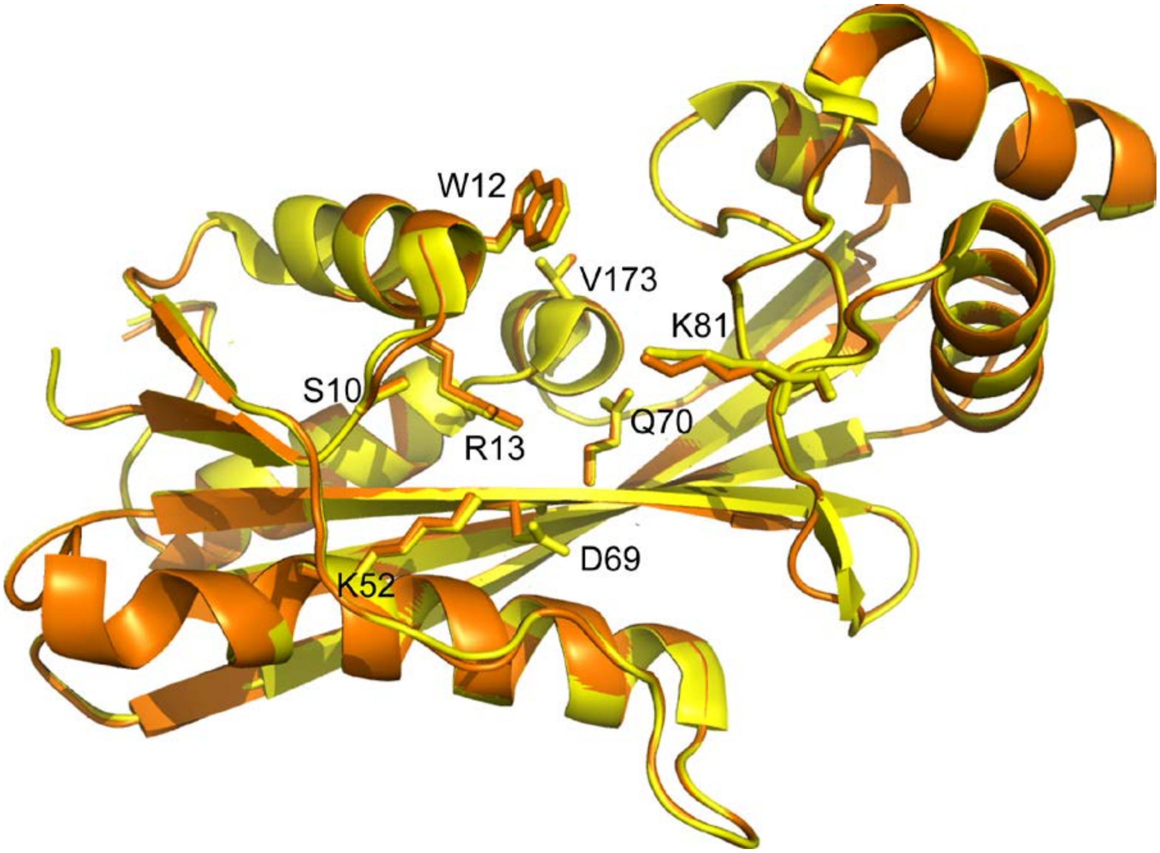

Figure S6, related to Figure 5. The pyrophosphate-like electron density found in the active site of BSU28050 (PDB code 4HEB). Total omit map was calculated with SFCHECK from CCP4 suite (contoured at 1 σ) and revealed an additional electron density in the same position as phosphate in the ASMTL-Maf structure (PDB code 2P5X).

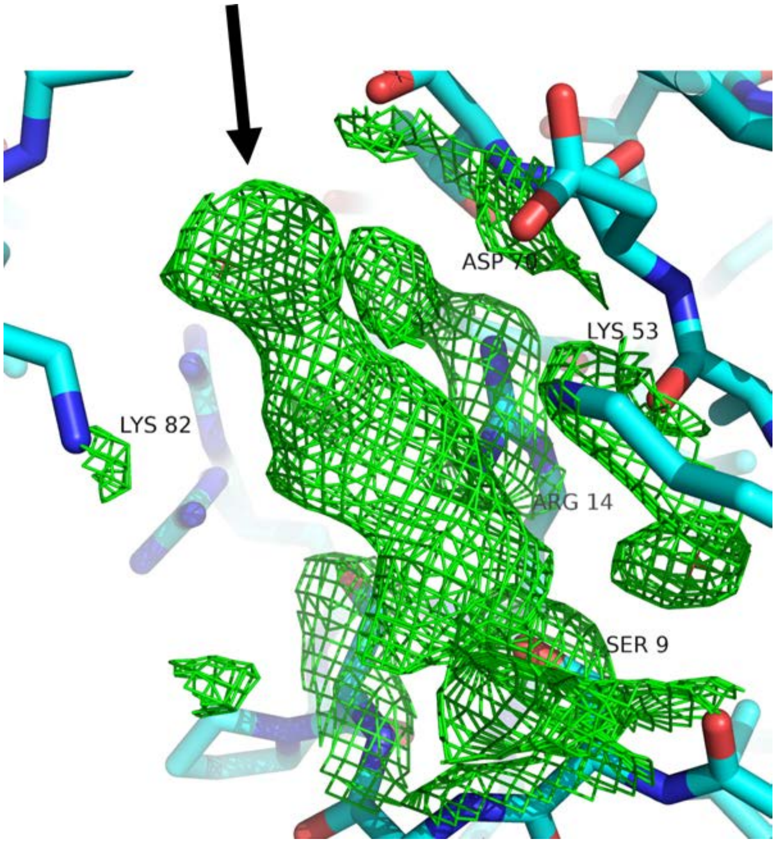

Figure S7, related to Figure 6. Structural basis for modified nucleotide binding in the active site of BSU28050. Manual docking of the modified nucleotide structures in the BSU28050 active site: (**A**), pseudoUTP; (**B**), m^5CTP; (**C**), CTP. (**A**), The recognition of pseudo-UTP can be mediated by the conserved bound water molecule (shown as the cyan sphere with H-bonds). (**B**), Binding of m^5CTP after a minor alteration of the Gln153 side chain conformation. (**C**), Unlike m^5CTP, the canonical cytosine base (turquoise) may be recognised in an alternative conformation resulting in non-productive binding of CTP in the active site.

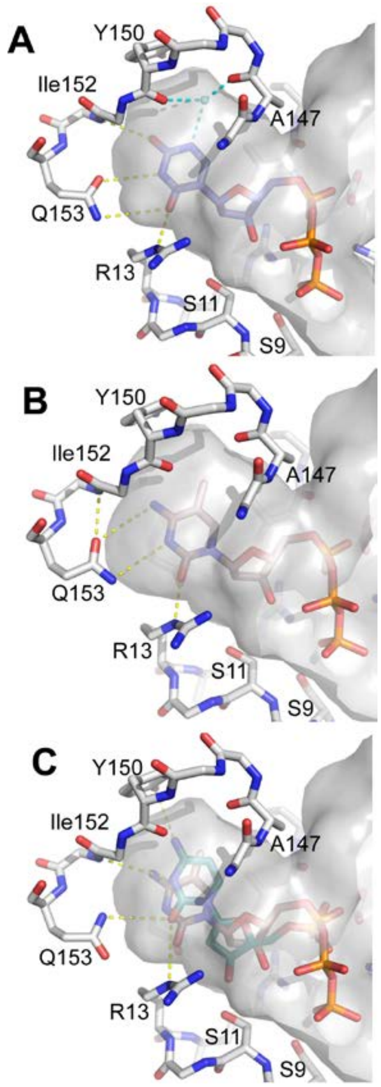

Supplement: Document S1. Figures S1–S7 and Tables S1 and S2 [file mmc1.pdf]
